# Comparison and Classification of LMW-GS Genes at *Glu-3* Loci of Common Wheat

**DOI:** 10.3390/genes16010090

**Published:** 2025-01-16

**Authors:** Yongying Zhao, Xianlin Zhao, Zhiguo Xiang, Dan Zhang, Hongshan Yang

**Affiliations:** Wheat Research Institute, Henan Academy of Agricultural Sciences (HAAS), Zhengzhou 450002, China; xlin918@126.com (X.Z.); xiangwheat@126.com (Z.X.); zhangdan20241212@163.com (D.Z.); yanghs20241212@163.com (H.Y.)

**Keywords:** common wheat, LMW-GS genes, *Glu-3* locus, sequence alignment, gene classification

## Abstract

Background: The low molecular weight glutenin subunits (LMW-GS) of wheat have great effects on food processing quality, but the resolution of LMW-GS and the scoring of their alleles by direct analysis of proteins are difficult due to the larger number of expressed subunits and high similarity of DNA sequences. It is important to identify and classify the LMW-GS genes in order to recognize the LMW-GS alleles clearly and develop the functional markers. Methods: The LMW-GS genes registered in GenBank were searched at NCBI, and 593 *Glu-3* genes with complete coding sequences were obtained, including 146 *Glu-A3*, 136 *Glu-B3*, and 311 *Glu-D3*. Sequence analysis and characterization of DNA and deduced amino acids were performed using the software DNAman. Results: The alignment and classification showed that there were at least 9 genes with 69 allelic variants at the *Glu-A3* locus, 11 genes with 64 allelic variants at the *Glu-B3* locus, and 10 genes with 96 variants at the *Glu-D3* locus, respectively. Furthermore, the specificity of some *Glu-3* genes and their variations was analyzed. Conclusions: The results were beneficial to understanding the LMW-GS genes fully and to developing the functional markers and will provide a theoretical reference for the quality improvement of wheat variety.

## 1. Introduction

Wheat flour has the distinctive quality properties to form the gluten network, which is suitable to make a lot of foods such as bread, steamed bread, noodles, cake, etc. [1,2]. Osborne first classified the grain proteins of wheat into four types based on their solubility, i.e., albumin dissolving in water or dilute buffer, globulin dissolving in salt solution, prolamin dissolving in 70–90% alcohol, and glutenin dissolving in dilute acid or dilute alkali [3]. The glutenin was further classified into high molecular weight subunits (HMW-GS) and low molecular weight subunits (LMW-GS), which contributed to the fundamental aspects of dough quality, for example, viscoelasticity and extensibility [4,5,6]. The proper viscoelasticity and extensibility were the basis to make good bread; thus, HMW-GS and LMW-GS alleles became the main targets of molecular marker-assisted selection in the quality improvement of wheat variety [7,8]. Because HMW-GS had a low gene copy number and clear resolution by gel electrophoresis, their allelic variation and relationship with wheat quality have been studied extensively [9,10,11], and the molecular markers based on polymerase chain reaction (PCR) were available to distinguish the important *Glu-1* alleles [12,13,14]. However, differentiating proteins and scoring alleles by direct analysis of LMW-GS were more difficult due to the larger number of expressed subunits and their overlapping mobility with the abundant gliadin proteins [15,16]. In view of this, the function of individual LMW-GS in the determination of wheat quality was less clear, though some alleles are clearly beneficial or detrimental [17,18,19]. It is very important to characterize LMW-GS genes and design functional markers for identifying different LMW-GS alleles [20].

Most LMW-GS are encoded by the complex *Glu-3* loci (*Glu-A3*, *Glu-B3*, and *Glu-D3*) on the short arms of group 1 chromosomes of wheat [21,22]. Gupta and Shepherd detected 20 different banding patterns of LMW-GS in bread wheat cultivars: 6 from *Glu-A3*, 9 from *Glu-B3*, and 5 from *Glu-D3* [23]. Twenty-six different LMW subunits were identified in one bread wheat cultivar according to the N-terminal amino acid sequences [24]. Based on the first N-terminal amino acid of mature protein, LMW-GS were classified into three types: LMW-m, LMW-s, and LMW-i, corresponding to methionine, serine, and isoleucine, respectively [25,26]. LMW-GS were further divided into 12 groups by Ikeda et al. according to deduced amino acid sequences and, in particular, the number and position of cysteine residues available for inter-molecular disulphide bond formation [27,28]. Long et al. (2005) retrieved 69 known LMW-GS genes from GenBank and classified them into nine groups by the deduced amino acid sequence of the highly conserved N-terminal domain [29]. Zhao et al. identified 6 LMW-GS genes, including 12 haplotypes at the *Glu-D3* locus, and made a clear distinction between the subunits, coding genes, alleles, and allelic variants (haplotypes) of LMW proteins for the first time [30,31]. Wang et al. cloned 6 *Glu-B3* genes, including 26 allelic variants, and 5 *Glu-A3* genes, including 19 allelic variations, respectively [32,33]. In a survey of the LMW-GS genes at NCBI (National Centre for Biotechnology Information, www.ncbi.nlm.nih.gov), a total of 1142 LMW-GS genes and gene fragments (partial genes) were obtained, of which there were 593 *Glu-3* genes with complete coding sequences and known loci, including 146 *Glu-A3* genes, 136 *Glu-B3* genes, and 311 Glu-D3 genes [6,27,34,35,36]. However, the detailed information of *Glu-3* genes and their allelic variations was somewhat mixed and confused due to the large number and high homology. In the paper, we try to make a thorough comparison between the *Glu-3* genes of common wheat and sort them out according to the similarity of DNA and deduced AA sequences, which will provide a theoretical reference for the quality improvement of wheat breeding.

## 2. Materials and Methods

The LMW-GS gene sequences used in the paper were all registered in GenBank, which were searched at NCBI (National Centre for Biotechnology Information, www.ncbi.nlm.nih.gov, accessed on 15 July 2024) by means of the *Glu-A3* genes, *Glu-B3* genes, and *Glu-D3* genes. All the 593 *Glu-3* gene accessions were listed in Table 1 and Table 2. Sequence analysis and characterization of DNA and deduced amino acids were performed using software DNAman V6.0.3.99 (http://www.lynnon.com).

## 3. Results

By searching in Genbank, a total of 1142 *Glu-3* genes and partial genes were obtained, of which there were 593 *Glu-3* genes with complete coding regions and known loci, including 146 *Glu-A3* gene sequences, 136 *Glu-B3* gene sequences, and 311 *Glu-D3* gene sequences. They were compared and grouped, respectively, based on their sequence variations of DNA and deduced amino acids. The results were as follows:

### 3.1. Composition and Variation of LMW-GS Genes at the Glu-D3 Locus

*Glu-D3* genes were first identified and classified systematically by Zhao et al. and a total of 6 *Glu-D3* genes with 12 allelic variations were characterized [30,31]. On this basis, the 311 *Glu-D3* genes with complete coding sequences from GenBank were compared by means of DNAman and grouped according to their similarity of DNA and deduced AA. The result showed that the *Glu-D3* locus contained at least 10 LMW-GS genes, including about 96 allelic variants (Table 1 and Table 2), of which the four newly sorted genes (*GluD3-7* to *GluD3-10*) presented a base similarity of ≤96.0% between each other and with the other six genes. The base similarity between allelic variants of each gene was presented in Table 2. Among the 10 genes, *GluD3-2* had the most abundant variations, including 31 at the base level or 25 at the AA level. Then *GluD3-1*, *GluD3-4*, and *GluD3-6* each had 11, 12, and 14 allelic variations, respectively. *GluD3-7* had the least allelic variation, and only one haplotype was found in GenBank. *GluD3-7*, *GluD3-9*, and *GluD3-10* were all pseudogenes, while *GluD3-4*, *GluD3-6*, and *GluD3-8* each had one or two pseudogene variants. In addition, a lot of duplicate sequences were found in GenBank, for example, 23 for *GluD3-1-1a*, 26 for *GluD3-2-1a*, 43 for *GluD3-4-2a*, 27 for *GluD3-5-1*, 30 for *GluD3-6-1a*, and 32 for *GluD3-9-1*.

**Table 1:** Homology comparison of the representatives of 10 *Glu-D3* genes (below diagonal) and their deduced amino acid sequences (above diagonal) (%, irrespective of the sequence length).

Accession	DQ35 7052	DQ35 7054	DQ35 7057	DQ45 7416	EU18 9096	DQ45 7420	EU18 9092	JQ32 0289	FJ75 5312	JF33 9168
DQ357052	100	78.9	78.3	82.4	77.5	78.5	77.6	78.8	-	-
DQ357054	79.9	100	87.9	87.9	82.7	82.9	87.6	83.5	-	-
DQ357057	78.1	88.0	100	82.7	86.7	82.2	87.6	81.8	-	-
DQ457416	89.4	89.0	87.2	100	82.3	85.7	84.3	86.3	-	-
EU189096	80.1	80.3	81.1	83.9	100	81.8	81.9	81.1	-	-
DQ457420	88.3	88.2	85.7	91.8	85.1	100	92.0	92.6	-	-
EU189092	84.8	93.0	91.2	89.3	81.0	93.4	100	86.7	-	-
JQ320289	88.0	89.2	84.7	91.1	84.2	95.9	91.7	100	-	-
FJ755312	87.3	88.3	84.3	90.7	84.1	95.0	90.5	96.0	100	-
JF339168	82.2	838	85.4	83.7	95.1	83.7	83.4	83.4	82.9	100

The representatives are the first allelic variant of each *GluD3* gene.

**Table 2:** Classification of 311 *Glu-D3* genes from GenBank.

Gene	Haplotype (Allelic Variation)		Notes	
	S/N	GenBank Accessions	DNA	AA
GluD3-1 ≧99.6%	1a	DQ357052(D3-11); FJ755313; JF339162; JX877785; JX877839; JX877858; JX877874; JX877927; JX877940; JX877958; JX877970; JX877986; JX878002; JX878018; JX878033; JX878065; JX878100; JX878172; JX878184; JX878203; KR612284; MG545991; MH347502;	23 same	350
	1b	JX877824;	1 single	
	2a	DQ357053(D3-12); EU189098; JX877890; JX877907; JX878049; JX878114; JX878126; JX878160; MG545992;	9 same	351
	2b	JX878145;	1 single	
	3–9	AB062865; AB062866; AB062867; KR612283; KR612285; KR612286; KR612287;	7 singles	321–350
GluD3-2 ≧99.3%	1a	DQ357054(D3-21); JX877783; JX877821; JX877838; JX877872; JX877905; JX877955; JX877968; JX877984; JX878000; JX878017; JX878032; JX878047; JX878063; JX878112; JX878124; JX878169; JX878200; KJ152532; KJ152533; KR612292; MH347500; KC222073; KC222075; KC222116; MN744871;	26 same	307
	1b–1d	JX877937; KJ152534; KJ152538;	3 singles	307
	2a	DQ357055(D3-22); JF339160; JX877804; JX877888; KC222089;	5 same	
	2b	KJ152537;	1 single	
	3a	DQ357056(D3-23); AB062875; FJ755315; FJ755322; JX877856; JX877925; JX878098; JX878181; MG545990; KC222110; KC222119; KC222121;	12 same	304
	3b–3c	AY299485; EU189094;	2 singles	
	4–24	AY263369; FJ172533; FJ615309; FJ615310; FJ615311; JQ320291; JQ796685; JQ796686; JQ796688; JQ796690; JX878083; KF020663; KF020664; KF020665; KJ152535; KJ152536; KR612288; KR612289; KR612290; KR612291; MG545996;	21 singles	304–308
	25	KJ152539;	1 single	Pseudo
GluD3-3 ≧98.4%	1	DQ357057(D3-31); JF339167; JX877790; JX877828; JX877841; JX877878; JX877894; JX877912; JX877944; JX877961; JX877976; JX877989; JX878006; JX878037; JX878069; JX878086; JX878206; KR612295; MG545995; MH347499; HQ619911; HQ619917;	22 same	354
	2	DQ357058(D3-32); FJ755316; JF339182; JF339199; JX877862; JX877929;	6 same	354
	3–7	EU189095; FJ755323; KR612293; KR612294; MG545994;	5 singles	354–383
GluD3-4 ≧98.2%	1	DQ457416(D3-41); JX878122;	2 same	304
	2a	DQ457417(D3-42); EU189093; KR612296; JF339158; JX877781; JX877802; JX877819; JX877836; JX877870; JX877886; JX877903; JX877923; JX877935; JX877953; JX877966; JX877982; JX877999; JX878015; JX878030; JX878045; JX878061; JX878081; JX878110; JX878142; JX878157; JX878167; JX878179; JX878198; MH347503; JF339174; MN744843; MN744846; MN744858; MN744861; MN744866; MN744869; MN744875; MN744877; MN744888; MN744895; MN744898; MN744904; MN744908;	43 same	303
	2b–2c	AB062872; MG545989;	2 singles	
	3–4	DQ457418(D3-43); JX877854;	2 singles	Pseudo
	5–10	FJ755314; HM055909; JQ320290; JQ796689; JX878096; R612297;	6 singles	299–303
GluD3-5 ≧98.4%	1	EU189096; FJ755310; JF339165; JX877789; JX877810; JX877827; JX877840; JX877861; JX877877; JX877893; JX877911; JX877943; JX877960; JX877975; JX877988; JX878005; JX878021; JX878036; JX878051; JX878068; JX878104; JX878148; JX878205; MG545993; MH347504; JF339181; JF339197;	27 same	365
	2–8	DQ457419(D3-5); AB062851; EU189097; FJ755317; JX878085; KR612298(97.3%); KR612299;	7 singles	345–365
GluD3-6 ≧98.3%	1a	DQ457420(D3-6); EU189091; JX877800; JX877816; JX877834; JX877851; JX877868; JX877884; JX877900; JX877920; JX877933; JX877950; JX877964; JX877979; JX877996; JX878012; JX878027; JX878042; JX878058; JX878079; JX878107; JX878119; JX878139; JX878154; JX878164; JX878176; JX878195; KR612300; MH347501; AB062873;	30 same	298
	1b	JF339155; JF339172; JF339203;	3 same	
	1c	KR612306; KR612307;	2 same	
	1d–1e	KR612302; KR612305;	2 singles	
	2–8	EU189090(98.6%); FJ755311; JX878094; KR612301; KR612303; KR612304; MG545988;	7 singles	
	9–10	FJ755318; JX877778;	2 singles	Pseudo
GluD3-7	1	EU189092;	1 single	288
GluD3-8 ≧99.0%	1–2	JQ320289; JQ796687;	2 singles	297
	3	FJ755319;	1 single	Pseudo
GluD3-9 ≧99.0%	1	FJ755312; KR612309; MG545941; JX877801; JX877818; JX877835; JX877853; JX877869; JX877885; JX877902; JX877922; JX877934; JX877952; JX877965; JX877981; JX877998; JX878014; JX878029; JX878044; JX878060; JX878080; JX878095; JX878109; JX878121; JX878141; JX878156; JX878166; JX878178; JX878197; JF339173; JF339188; JX828371;	32 same	Pseudo
	2–4	JF339157; JX877780; KR612310;	3 singles	
GluD3-10 100%	1	JF339168; JX877812; JX877843; JX877864; JX877990; JX878087; MG545944;	7 same	Pseudo
	2	JX877829; JX877879; JX877896; JX877913; JX877930; JX877946; JX877962; JX878008; JX878023; JX878039; JX878070; MG545943;	12 same	
	3	JX878053; JX878128; MG545945;	3 same	
	4	JX878116; JX878151; MG545946;	3 same	
	5	MG545942; JX877792;	2 same	

The framed were genes located in the study by alignment.

### 3.2. Composition and Variation of LMW-GS Genes at the Glu-B3 Locus

The 136 *Glu-B3* genes from GenBank were aligned by DNAman and classified based on the study of Wand et al. [32]. In addition to the 7 LMW-GS genes with 26 variants known, another 4 genes (*GluB3-8* to *GluB3-11*) were grouped, which presented the similarity of ≤96.6% between each other and with others (Table 3 and Table 4). The base similarity between allelic variants of each gene was presented in Table 4.

Among the 11 genes, *GluB3-4* had the most abundant allelic variants, including 20 at the base level or 17 at the AA level. On the contrary, *GluB3-7* had the least, and only one pseudogene was found in GenBank. The very high similarities were found between *GluB3-1* and *GluB3-5* (99.7%) and between *GluB3-3* and *GluB3-6* (99.8%). It was interesting that 24 duplicate sequences of the *GluB3-7* haplotype were found in GenBank, the most for *Glu-B3* genes, suggesting that *GluB3-7* was more stable.

### 3.3. Composition and Variation of LMW-GS Genes at the Glu-A3 Locus

The 146 *Glu-A3* genes with complete coding sequences from GenBank were also compared with each other and were grouped based on the study of Wang et al. [33], who identified 5 *Glu-A3* genes with 19 allelic variations. The result showed that the *Glu-A3* locus contained at least 9 LMW-GS genes, including about 69 allelic variants, of which the four newly sorted genes (*GluA3-6* to *GluA3-9*) showed a base similarity of ≤96.8% (Table 5 and Table 6). The base similarity between allelic variants of each gene was ≧98.9% for *GluA3-1*, ≧98.2% for *GluA3-2*, ≧99.6% for *GluA3-3*, ≧99.2% for *GluA3-4*, ≧99.8% for *GluA3-6*, and ≧98.6% for *GluA3-8*.

Among the 9 genes, *GluA3-1* had the most abundant variations, including 22 at the base level or 20 at the AA level. *GluA3-2* also had 13 allelic variations, including three pseudogene variations. GluA3-3 had six allelic variants, but all were pseudogenes. *GluA3-5*, *GluA3-7*, and *GluA3-9* presented the least allelic variation.

## 4. Discussion

### 4.1. Characteristics of LMW-GS Genes and Their Deduced AA Sequences

LMW-GS accounts for about one third of the seed storage proteins and has great effects on the end-use quality of wheat [7,8]. Thus, they have received considerable attention from wheat researchers all the time [37]. The sequence analysis showed that the coding regions of LMW-GS genes were not interrupted by introns and were highly conserved at 5′- and 3′-terminal sequences [31]. Each haplotype encoded a highly conserved signal peptide of 20 amino acids and a short N-terminal conserved region with 13 amino acids, followed by an N-terminal repetitive domain and then a C-terminal conserved domain involving three sub-regions of cysteine-rich, glutamine-rich, and final conserved domain. The longest and the shortest LMW glutenin subunits at the *Glu-A3*, *Glu-B3*, and *Glu-D3* loci were 391/212, 393/257, and 383/288 amino acids, respectively, indicating that the length change of *Glu-D3* genes was relatively small. All the deduced LMW-GS showed a typical eight conserved cysteine residues except for a few mutations [38], which were the same as the B-hordeins of barley [39]. It is very useful to characterize the LMW-GS genes because of the difficulties in differentiating the proteins by SDS-PAGE [20].

### 4.2. Classification and Specificity of LMW-GS Genes at Three Glu-3 Loci

A lot of LMW-GS genes have been cloned and registered in GenBank [34,35,36], but the relationship of them with each other was not very clear. In view of this, 593 *Glu-3* genes with complete coding sequences (some with partial or without signal peptide) were obtained from GenBank and compared by means of DNAman, of which the 146 *Glu-A3* genes were classified into 9 groups with 69 variations, the 136 *Glu-B3* genes were classified into 11 groups with 64 variations, while the 311 *Glu-D3* genes were classified into 10 groups with 96 variations. In addition, 47 LMW-GS gene sequences, which loci were unknown before, were newly located in the study, including 7 *Glu-A3*, 5 *Glu-B3,* and 35 *Glu-D3* genes, because they had 100% base similarity with the related genes (Table 2, Table 4 and Table 6, framed). Obviously, the *Glu-D3* locus had the most abundant LMW-GS haplotypes and allelic variations [26]. The similarities of genes were higher between the allelic variants of each gene and relatively lower between the classified groups, but some exceptions were found in the study. For example, there was 99.7% similarity between *GluB3-1* (EU369699) and *GluB3-5* (EU369706) haplotypes and 99.8% between *GluB3-3* (EU369715) and *GluB3-6* (EU369711) haplotypes (Table 3), respectively, which were previously classified by Wang et al. [32], indicating that they might have the same origin, no matter at the gene level or variant level. The results indicated that the LMW-GS genes were more complicated than expected and worthy of being studied further.

### 4.3. Comparison of LMW-GS Genes Between Glu-A3, Glu-B3, and Glu-D3 Loci

Most of the LMW-GS genes were jointly encoded by the *Glu-A3*, *Glu-B3*, and *Glu-D3* loci, which were located on the short arms of the A1, B1, and D1 wheat chromosomes, respectively [21,22,40]. However, the relationships of LMW-GS genes between the three *Glu-3* loci were not very clear, so we carried out a horizontal comparison between *Glu-A3*, *Glu-B3*, and *Glu-D3* genes. In most cases, the base similarities were lower than 90% between the *Glu-A3*, *Glu-B3*, and *Glu-D3* genes, but there were some exceptions in the study. For example, the base similarity between *GluA3-7*, *GluB3-8*, and *GluD3-1* reached 99.1%, 99.2%, and 99.7%, respectively (Supplementary Tables S1–S3), while the similarity of *GluA3-6* with *GluD3-2* reached 100%. The results interpreted why it was difficult to differentiate the three *Glu-3* proteins by SDS-PAGE [15,16] and also presented the importance of characterizing the LMW-GS genes and their allelic variations [20].

## 5. Conclusions

In summary, 593 complete LMW-GS genes at *Glu-3* loci were obtained from GenBank. They were compared systematically by means of DNAman and classified based on their similarities of base and AA sequences. It was found that there were at least 9 genes with 69 allelic variants, 11 genes with 64 allelic variants, and 10 genes with 96 variants, respectively, at the *Glu-A3*, *Glu-B3*, and *Glu-D3* loci. The base similarities between the *Glu-3* genes were generally less than 90%, although the highest could reach to over 99.8%, presenting the specificity and complexity of *Glu-3* gene composition. The results are beneficial to understanding the *Glu-3* genes comprehensively and will provide a theoretical basis for developing functional markers of LMW-GS genes and for characterizing the LMW-GS genes of wheat-related species such as *Triticum monococcum*, *Triticum dicoccum*, *Aegilops tauschii*, etc.

## Figures and Tables

**Table 3:** Homology comparison of the representatives of 11 *Glu-B3* genes (below diagonal) and their deduced amino acid sequences (above diagonal) (%, irrespective of the sequence length).

Accession	EU36 9699	EU36 9704	EU36 9715	EU36 9724	EU36 9706	EU36 9711	EU36 9731	DQ63 0441	KF02 0660	JF33 9166	JX87 7826
EU369699	100	93.8	93.9	85.3	100	93.9	-	77.9	75.5	-	83.4
EU369704	95.0	100	92.1	84.2	93.8	92.1	-	78.2	75.9	-	82.7
EU369715	94.6	94.5	100	84.9	93.9	100	-	77.0	75.9	-	83.1
EU369724	887	88.9	89.5	100	85.3	84.9	-	79.0	75.4	-	94.9
EU369706	99.7	95.2	94.8	88.4	100	93.9	-	77.9	75.5	-	83.4
EU369711	94.8	94.3	99.8	89.0	95.2	100	-	77.0	75.9	-	83.1
EU369731	88.2	89.2	89.1	95.2	88.1	88.9	100	-	-	-	-
DQ630441	82.7	83.3	82.2	84.0	82.5	82.0	83.6	100	90.8	-	77.7
KF020660	82.3	83.1	81.8	82.8	82.1	81.6	82.1	95.4	100	-	74.8
JF339166	95.5	95.1	96.0	89.1	95.8	96.0	88.9	81.9	81.2	100	-
JX877826	87.9	89.0	89.0	96.6	88.0	89.0	95.8	83.3	82.0	89.4	100

The representatives are the first allelic variant of each GluB3 gene.

**Table 4:** Classification of 136 *Glu-B3* genes from GenBank.

Gene	Haplotype (Allelic Variation)		Notes	
	S/N	GenBank Accessions	DNA	AA
GluB3-1 ≧99.2%	1–6	EU369699(B3-11); EU369700(B3-12); EU369701(B3-13); EU369702(B3-14); EU369703(B3-15); MH347497;	6 singles	343–365
GluB3-2 ≧99.4%	1	EU369704(B3-21); MH347496;	2 same	370
	2	EU369721(B3-22); EU369722; EU369723;	3 same	369
	3–5	EU369705(B3-23); JX163861, JX163862,	3 singles	369
GluB3-3 ≧99.6%	1	EU369717(B3-33); AB119006;	2 same	392
	2–6	EU369715(B3-31); EU369716(B3-32); EU369718(B3-34), FJ755309; KR612277;	5 singles	392
GluB3-4 ≧98.9%	1	EU369724(B3-41); EU369725; EU369726; JF339163; JX877971; JX878003; JX878019; JX878034; JX878050; JX878066; JX878101; JX878146; MH347498; JF339194;	14 same	350
	2a	EU369719(B3-42); JX877806; JX877845; JX877875; JX877908; JX877928; JX878127; KR612281; HQ619905; JF339179;	10 same	350
	2b	EU369727(B3-43); EU369728;	2 same	
	3a	EU369729(B3-44); EU369730; JX877786; JX877859; JX878134; JX878161;	6 same	350
	3b	EU189089;	1 single	
	4a	JX878115;	1 single	350
	4b	JX878212;	1 single	
	5	JX877823; JX877939; JX877957; JX878091; JX878171; JX878183; JX878191; JX878202;	8 same	343
	6–17	EU369720(B3-45); AB062852; FJ755306; FJ876823; FJ876824; JX877891; FJ876825; FJ972196; KR612278; KR612279; KR612280; KR612282;	12 singles	269–350
GluB3-5 ≧99.6%	1–4	EU369706(B3-51); EU369707(B3-52); EU369708(B3-53); EU369709(B3-54);	4 singles	360–365
	5	EU369710(B3-55); EU189088; AB262661;	3 same	343
GluB3-6 ≧99.2%	1–6	EU369711(B3-61); EU369712(B3-62); EU369713(B3-63); EU369714(B3-64); JX877832; JX878089;	6 singles	392–393
GluB3-7 ≧99.7%	1	EU369731(GluB3-71); EU369732; EU369733; EU369734; EU369735; EU369736; JF339164; JX877788; JX877807; JX877825; JX877860; JX877876; JX877892; JX877909; JX877941; JX878004; JX878020; JX878035; JX878067; JX878084; JX878147; JF339180; JF339196; JF271919;	24 same	Pseudo
	2–3	JX877972; JX878204;	2 singles	
GluB3-8 ≧99.9%	1	DQ630441; KJ152530;	2 same	349 257–350 Pseudo
	2–6	DQ630442; KF020661; KF020662; KJ152528; KJ152531;	5 singles	
	7	KJ152529;	1 single	
GluB3-9	1	KF020660;	1 single	350
GluB3-10 ≧99.8%	1	JF339166; JX877811; JX877842; JX877895; JX877945; JX878007; JX878022; JX878038; JX878052; JX878135; JX878149;	11 same	Pseudo
	2	JF339198; JX877791; JX877863; JX878213;	4 same	
	3	JX878173;	1 single	
GluB3-11	1a	JX877826; JX877959;	1 same	364
	1b	JX877942;	1 single	

The framed were genes located in the study by alignment.

**Table 5:** Homology comparison of the representatives of eight *Glu-A3* genes (below diagonal) and their deduced amino acid sequences (above diagonal) (%, irrespective of the sequence length).

Accession	FJ54 9928	FJ54 9937	FJ54 9939	FJ54 9945	FJ54 9946	MG57 4323	DQ63 0440	JF33 9156	FJ75 5305
FJ549928	100	71.2	-	96.2	72.3	74.3	68.8	-	-
FJ549937	83.5	100	-	69.1	97.7	88.2	82.2	-	-
FJ549939	93.2	84.3	100	-	-	-	-	-	-
FJ549945	94.0	82.9	93.2	100	70.8	72.2	68.4	-	-
FJ549946	83.6	98.7	84.5	82.9	100	87.2	83.1	-	-
MG574323	84.5	91.2	84.7	83.4	90.6	100	80.3	-	-
DQ630440	82.3	88.4	83.3	82.0	87.7	96.2	100	-	-
JF339156	84.0	94.3	84.2	82.8	93.9	91.8	88.4	100	-
FJ755305	94.7	82.4	93.6	96.8	82.2	84.7	81.3	82.9	100

The representatives are the first allelic variant of each *GluA3* gene.

**Table 6:** Classification of 146 *Glu-A3* Genes from GenBank.

Gene	Haplotype (Allelic Variation)		Notes	
	S/N	GenBank Accessions	DNA	AA
GluA3-1 ≧98.9%	1	FJ549928(A3-11); AY453154; JF339169; JX878117; MH347495;	5 same	376
	2	FJ549929(A3-12); AY453155;	2 same	384
	3a	FJ549930(A3-13); AY453156; JX877793; JX877830; JX877865; JX877963; JX878075; JX878152; JX878174; JX878207; JX878214; JF339201;	12 same	376
	3b	KJ152523;	1 single	
	4–6	FJ549931(A3-14); FJ549932(A3-15); FJ549933(A3-16);	3 singles	356
	7a	FJ549934(A3-17); EU189087;	2 same	388
	7b	AY453160;	1 single	
	8–20	AB062876; AY453157; AY453158; AY453159; EU871816; FJ755304; FJ876819; FJ876820; FJ876821; FJ876822; KR612275; KR612276; KC136287	13 singles	212–388
GluA3-2 ≧98.2%	1–3	FJ549935(A3-21); FJ549936(A3-22); KR612308;	3 singles	Pseudo
	4	FJ549937(A3-23); JX877803; JX878097;	3 same	304
	5	FJ549938(A3-24); JX877837; JX877871; JX877887; JX877936; JX877967; JX878218;	7 same	306
	6	JX877782; JX877855; JX877904; JX877924; JX878016; JX878031; JX878046; JX878062; JX878123; JX878143; JX878168; JX878180; JX878210; JX878221;	14 same	Pseudo
	7	X877820; X877983; X878224;	3 same	Pseudo
	8	JX878082; JX878132; JX878189;	3 same	307
	9–15	AB062868; AB062869; AB062870; AB062871; FJ755302; JQ320288; JQ320292;	7 singles	279–304
GluA3-3 ≧99.6%	1	FJ549939(A3-31); JF339161; JX877784; JX877956; JX878113; JX878144; JX878159;	7 same	Pseudo
	2	FJ549940(A3-32); JX877994;	2 same	
	3–4	FJ549941(A3-33); FJ549944(A3-36);	2 singles	
	5	FJ549942(A3-34); JX878125; JX878133;	3 same	
	6	FJ549943(A3-35); JX877906; JX878182;	3 same	
	7	JX877822; JX878170; JX878201; JX878211;	4 same	
GluA3-4 ≧99.2%	1a	FJ549945(A3-4);	1 single	390
	1b	FJ755303; JX877815; JX877850;	3 same	
	2–9	AB062877; AB062878; DQ630443; JX877798; JX877977; JX878105; KC136285; KC136286;	8 singles	389–391
GluA3-5	1	FJ549946(A3-5);	1 single	301
GluA3-6 ≧99.8%	1a	MG574323; MN744845; MN744855; MN744864; MN744897;	5 same	307
	1b	MG574327;	1 single	
	2–6	MG574321; MG574322; MG574324; MG574325; MG574326;	5 singles	
GluA3-7	1–2	DQ630440; MG574328;	2 singles	350
GluA3-8 ≧98.6%	1	JF339156; JX877817; JX877852; JX877921; JX877951; JX877980; JX877997; JX878013; JX878028; JX878043; JX878059; X878108; JX878120; JX878140; JX878155; JX878165; JX878177; X878196;	18 same	pseudo
	2	JX877799; JX878093	2 same	
	3	JX877833; JX877867; JX877883	3 same	
	4	JX877901	1 single	
GluA3-9	1	FJ755305;	1 single	pseudo

The framed were located in the study by alignment.

## Data Availability

The original contributions presented in this study are included in the article/Supplementary Materials. Further inquiries can be directed to the corresponding author.

## Supplementary Materials

The following supporting information can be downloaded at: https://www.mdpi.com/article/10.3390/genes16010090/s1, Table S1: Homology comparison of the representatives of LMW-GS genes between Glu-A3 and Glu-B3 loci (%, irrespective of the sequence length); Table S2: Homology comparison of the representatives of LMW-GS genes between Glu-A3 and Glu-D3 loci (%, irrespective of the sequence length); Table S3: Homology comparison of the representatives of LMW-GS genes between Glu-A3 and Glu-D3 loci (%, irrespective of the sequence length).
