# Supplementary material for: Comparison and Classification of LMW-GS Genes at Glu-3 Loci of Common Wheat
Source: Genes (Basel). 2025 Jan 16;16(1):90. doi: 10.3390/genes16010090 (PMC11765225; doi:10.3390/genes16010090)
Supplement: Supplementary file 1 [file genes-16-00090-s001.zip › genes-3395432-supplementary.pdf]

**Table S1.** Homology comparison of the representatives of LMW-GS genes between Glu-A3 and Glu-B3 loci (%, irrespective of the sequence length).

| Gene S/N | A3-1 | A3-2 | A3-3 | A3-4 | A3-5 | A3-6 | A3-7 | A3-8 | A3-9 |
|---|---|---|---|---|---|---|---|---|---|
| GluB3-1 | 76.4 | 73.9 | 76.0 | 76.2 | 73.4 | 81.8 | 71.2 | 78.3 | 79.5 |
| GluB3-2 | 80.5 | 75.1 | 82.2 | 79.7 | 74.4 | 84.7 | 71.2 | 81.7 | 80.4 |
| GluB3-3 | 79.7 | 75.0 | 82.2 | 78.6 | 74.4 | 83.0 | 71.7 | 80.8 | 79.1 |
| GluB3-4 | 67.2 | 86.6 | 68.4 | 66.9 | 86.0 | 77.1 | 84.7 | 86.8 | 70.1 |
| GluB3-5 | 79.4 | 74.0 | 81.7 | 78.9 | 73.4 | 81.7 | 71.5 | 80.4 | 78.4 |
| GluB3-6 | 79.5 | 74.9 | 82.3 | 78.4 | 74.2 | 71.7 | 81.1 | 81.1 | 78.6 |
| GluB3-7 | 77.9 | 84.4 | 80.6 | 78.2 | 84.4 | 83.8 | 81.8 | 85.2 | 77.6 |
| GluB3-8 | 80.1 | 88.1 | 82.8 | 80.8 | 87.8 | 87.1 | 99.1 | 89.0 | 79.8 |
| GluB3-9 | 81.5 | 86.3 | 83.9 | 83.0 | 85.9 | 83.8 | 94.0 | 86.6 | 81.9 |
| GluB3-10 | 80.0 | 79.1 | 80.1 | 79.9 | 78.5 | 82.0 | 78.1 | 80.1 | 79.9 |
| GluB3-11 | 77.7 | 80.0 | 77.9 | 77.8 | 79.3 | 79.1 | 79.8 | 81.3 | 78.4 |

The representatives are the first allelic variant of 9 GluA3 and 11 GluB3 genes.

**Table S2.** Homology comparison of the representatives of LMW-GS genes between Glu-A3 and Glu-D3 loci (%, irrespective of the sequence length).

| Gene S/N | A3-1 | A3-2 | A3-3 | A3-4 | A3-5 | A3-6 | A3-7 | A3-8 | A3-9 |
|---|---|---|---|---|---|---|---|---|---|
| GluD3-1 | 80.3 | 88.5 | 81.3 | 79.9 | 88.2 | 86.3 | 99.2 | 89.4 | 77.2 |
| GluD3-2 | 84.8 | 90.9 | 84.4 | 83.8 | 90.4 | 100 | 86.5 | 91.4 | 80.1 |
| GluD3-3 | 84.3 | 86.8 | 84.6 | 83.0 | 86.4 | 88.3 | 83.2 | 87.0 | 80.2 |
| GluD3-4 | 82.7 | 94.5 | 82.4 | 82.1 | 94.1 | 91.6 | 87.2 | 94.5 | 80.8 |
| GluD3-5 | 79.5 | 87.1 | 81.7 | 80.2 | 87.1 | 85.4 | 86.2 | 87.3 | 79.1 |
| GluD3-6 | 82.2 | 90.0 | 82.0 | 81.8 | 89.6 | 86.6 | 85.2 | 88.2 | 79.9 |
| GluD3-7 | 84.5 | 88.2 | 84.4 | 84.2 | 87.7 | 89.2 | 84.1 | 86.3 | 81.1 |
| GluD3-8 | 80.6 | 89.5 | 79.9 | 80.0 | 89.2 | 87.8 | 86.0 | 88.4 | 80.2 |
| GluD3-9 | 80.0 | 89.2 | 79.6 | 79.3 | 89.1 | 87.7 | 85.7 | 88.1 | 79.6 |
| GluD3-10 | 75.7 | 77.4 | 76.0 | 75.2 | 77.6 | 77.3 | 77.7 | 78.9 | 75.1 |

The representatives are the first allelic variant of 9 GluA3 and 10 GluD3 gene.

**Table S3.** Homology comparison of the representatives of LMW-GS genes between Glu-A3 and Glu-D3 loci (%, irrespective of the sequence length).

| Gene S/N | B3-1 | B3-2 | B3-3 | B3-4 | B3-5 | B3-6 | B3-7 | B3-8 | B3-9 | B3-10 | B3-11 |
|---|---|---|---|---|---|---|---|---|---|---|---|
| GluD3-1 | 72.3 | 67.6 | 68.9 | 59.0 | 72.0 | 69.3 | 68.9 | 99.7 | 95.1 | 73.6 | 74.7 |
| GluD3-2 | 73.7 | 70.9 | 71.1 | 61.1 | 75.3 | 73.3 | 68.1 | 86.0 | 83.8 | 77.8 | 74.5 |

| | | | | | | | | | | | |
|---|---|---|---|---|---|---|---|---|---|---|---|
| GluD3-3 | 95.6 | 96.1 | 95.8 | 76.9 | 95.9 | 95.8 | 88.1 | 69.7 | 69.3 | 96.9 | 88.2 |
| GluD3-4 | 70.5 | 68.4 | 67.7 | 66.7 | 72.1 | 70.0 | 67.9 | 88.9 | 87.4 | 72.7 | 73.7 |
| GluD3-5 | 88.2 | 88.1 | 89.1 | 95.7 | 87.6 | 88.5 | 95.5 | 82.3 | 81.1 | 88.7 | 96.6 |
| GluD3-6 | 84.6 | 85.8 | 85.1 | 86.3 | 84.5 | 84.7 | 85.6 | 85.7 | 83.6 | 85.4 | 85.8 |
| GluD3-7 | 88.8 | 89.0 | 88.1 | 78.1 | 89.0 | 88.2 | 84.7 | 84.9 | 83.0 | 89.6 | 84.6 |
| GluD3-8 | 77.1 | 76.3 | 75.6 | 76.6 | 77.1 | 75.6 | 75.8 | 79.1 | 78.2 | 74.7 | 76.8 |
| GluD3-9 | 77.1 | 76.4 | 75.7 | 76.1 | 77.1 | 75.7 | 75.3 | 78.9 | 78.0 | 74.5 | 76.7 |
| GluD3-10 | 85.3 | 86.5 | 86.5 | 95.7 | 85.2 | 86.6 | 95.5 | 81.4 | 80.2 | 86.6 | 93.6 |

The representatives are the first allelic variant of 11 GluB3 and 10 GluD3 genes.
